# Hyperglycemia‐Induced Microglial CTSS Disrupts Blood‐Retinal Barrier Integrity via PAR2‐Dependent Downregulation of Endothelial MFSD2A

**DOI:** 10.1002/cns.71187

**Published:** 2026-09-28

**Authors:** Chengye Xu, Hongxue Li, Qian Xu, Kangqi Zhao, Yangwen Chen, Yan Wang, Wei Zou, Binglin Kuang, Hongyu Kuang

**Affiliations:** ^1^ The Department of Endocrinology The First Affiliated Hospital of Harbin Medical University Harbin China; ^2^ Acupuncture and Moxibustion Department III The First Affiliated Hospital of Heilongjiang University of Traditional Chinese Medicine Harbin China

**Keywords:** Cathepsin S, diabetic retinopathy, endothelial cells, major facilitator superfamily domain‐containing protein 2a, microglia

## Abstract

**Background:**

Diabetic retinopathy (DR), a leading cause of blindness in diabetes, involves dysregulated neuroimmune crosstalk. While microglial activation and endothelial dysfunction are established in DR, the molecular mechanisms linking innate immunity to blood‐retinal barrier (BRB) breakdown remain elusive.

**Methods:**

We combined single‐cell RNA sequencing (scRNA‐seq) of diabetic mouse retinas with in vivo models, retinal endothelial MFSD2A overexpression, and microglial Cathepsin S (CTSS) knockdown via adeno‐associated virus (AAV). Human retinal microvascular endothelial cells (HRMECs) and human microglial cells (HMC3) were subjected to high‐glucose conditions combined with in vitro systems (transwell co‐cultures, holographic 3D tomography). Mechanistic studies employed siRNA knockdown, plasmid overexpression, exogenous CTSS supplementation, and transcytosis assays.

**Results:**

scRNA‐seq revealed diabetes‐induced CTSS upregulation in retinal microglia (*p* = 0.0031) and MFSD2A downregulation in endothelial cells (*p* = 0.01). Microglial CTSS promoted M1 polarization (*p* < 0.001) and pro‐inflammatory cytokine secretion (IL‐1β and TNF‐α, *p* < 0.05), while Ctss knockdown attenuated these effects (*p* < 0.05). CTSS activated endothelial PAR2, suppressing endothelial MFSD2A and enhancing caveolin‐1‐mediated transcytosis. Endothelial MFSD2A overexpression reduced vascular permeability independently of tight junction modulation (ZO‐1, Occludin, Claudin‐5, *p* > 0.05). Microglia‐endothelial crosstalk was disrupted in diabetes, with ultrastructural alterations (cytoplasmic shrinkage, microglial amoeboid transformation) promoting cytotoxic interactions and exacerbate vascular endothelial damage.

**Conclusions:**

Hyperglycemia induces retinal microglial activation and CTSS upregulation, disrupting microglia‐endothelial crosstalk via the PAR2 pathway, suppressing endothelial MFSD2A expression, enhancing transcytosis, compromising iBRB integrity, and accelerating DR progression. Therapeutic strategies targeting microglial CTSS or endothelial MFSD2A represent promising avenues for preserving vision in diabetic patients.

## Graphical abstract

Hyperglycemia induces retinal microglial activation and CTSS upregulation, disrupting microglia‐endothelial crosstalk via PAR2‐dependent MFSD2A suppression. Abnormal activation of microglia amplifies retinal inflammatory cascade, enhances transcytosis, compromises BRB integrity, and accelerates DR progression.

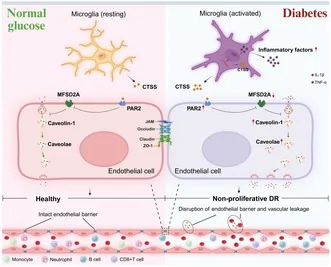

## 1 Introduction

Diabetic retinopathy (DR), a prevalent microvascular complication of diabetes, represents a leading cause of preventable vision loss and blindness among the working‐age population [1]. Beyond its ocular manifestations, DR significantly elevates risks of including all‐cause mortality and cardiovascular disease‐related deaths [2]. Current projections estimate that the global burden of vision impairment attributable to DR will escalate to 700 million cases by 2045, underscoring its escalating public health impact [3]. As a multifactorial disorder, DR pathogenesis involves intricate interactions between genetic predisposition, metabolic dysregulation, and inflammatory cascades. Genome‐wide association studies and experimental models have highlighted the pivotal role of inflammatory pathways, particularly those mediated by retinal innate immune cells, in driving disease progression [2, 4].

The innate immune system serves as a critical modulator of retinal immune‐inflammatory equilibrium, with microglia acting as resident “immune sentinels” to maintain microenvironmental homeostasis [5, 6]. Under diabetic conditions, aberrant microglial activation disrupts this balance, triggering endothelial dysfunction through maladaptive crosstalk between microglia and vascular endothelial cells. This interplay constitutes a key mechanism underlying the transition from preclinical vascular abnormalities to clinically overt DR. Central to this process is the blood‐retinal barrier (BRB), a structural and functional safeguard that isolates retinal tissue from systemic circulation while preserving immune privilege [7]. BRB integrity relies heavily on regulated transcytosis, a vesicle‐mediated transport system controlling endothelial permeability. Major facilitator superfamily domain‐containing protein 2a (MFSD2A), a transcytosis inhibitor enriched in microvascular endothelial cells, plays a critical role in maintaining BRB stability through suppressing caveolin (CAV)‐dependent pathways [8]. Notably, diabetic retinas exhibit diminished MFSD2A+ endothelial populations alongside upregulated inflammatory genes in microglia, suggesting a mechanistic link between innate immune activation and vascular leakage [9]. However, the molecular drivers of microglia‐endothelial communication and their impact on MFSD2A‐mediated permeability remain poorly defined.

Cathepsin S (CTSS), a lysosomal cysteine protease overexpressed in inflammatory diseases [10], plays dual roles in immune regulation, facilitating adaptive immunity through MHC Class II (MHC‐II) maturation via invariant chain cleavage, enabling antigen presentation with co‐stimulatory molecules and promoting peripheral immune cell activation and retinal infiltration via innate immune pathways [10, 11, 12]. In this study, we identified CTSS, a lysosomal cysteine protease upregulated in diabetic microglia, as a pivotal regulator of retinal immunoinflammatory homeostasis. Single‐cell RNA sequencing in a diabetic mouse model revealed CTSS overexpression in activated microglia, which was further validated through in vivo and in vitro experiments. We demonstrated that hyperglycemia‐induced CTSS exacerbates transcytosis via the endothelial protease activated receptor 2 (PAR2)/MFSD2A axis, disrupting vascular integrity and accelerating pathological progression from subclinical DR to advanced stages. These findings elucidate a novel CTSS‐PAR2‐MFSD2A signaling cascade that bridges microglial inflammation to endothelial dysfunction, providing potential therapeutic targets to mitigate diabetic retinal damage.

## 2 Methods

### 2.1 Establishment of Daibetic Mouse Model

Male C57BL/6J mice aged 6–8 weeks (20–23 g, Vital River Laboratories, Beijing) were purchased and housed under a 12 h light–dark cycle, 18°C–22°C.

After acclimatization, mice were randomly divided into two groups: Control (*n* = 40) and DR group (*n* = 60). DR group was fasted overnight (8–10 h) and then intraperitoneally injected with streptozotocin (STZ, Biotopped, Beijing) to induce daibetic mouse model: 0.1 mol/L citric acid (Tianda Chemical Reagent Factory, Tianjin) and trisodium citrate solutions (Tianda Chemical Reagent Factory, Tianjin) were mixed to prepare citrate buffer solution (pH 4.2–4.5). 1% STZ solution was prepared under dark on ice. DR group was injected intraperitoneally with 50 mg/kg STZ solution for five consecutive days, Control group with an equivalent volume of citrate buffer solution [3]. Mice with random blood glucose ≥ 16.7 mmol/L were confirmed the successfully diabetic model and included [3]. Samples were collected after 4 weeks of diabetes for subsequent analysis.

### 2.2 Intravitreal Injection of Adeno‐Associated Virus (AAV)

After acclimatization, mice were randomly divided into: Control group (*n* = 30), DR group (*n* = 40), MFSD2A negative control group (MFSD2A‐NC, *n* = 40), MFSD2A overexpression group (MFSD2A‐OE, *n* = 40), CTSS negative control group (CTSS‐NC, *n* = 40), and CTSS knock‐down group (CTSS‐KD, *n* = 40). diabetic mice model was established as described above.

We then performed intravitreal injection of AAV (Aopobio, Shanghai) in diabetic mice. Mice were anesthetized, dilated the pupils for 3–5 min, the eye surface was locally anesthetized with lidocaine ointment for 3–5 min, and kept the eye surface moist. Stabilized the eyeball with ophthalmic forceps and injected 2 μL of synthetic virus or control virus into the vitreous cavity 2 mm behind the corneal edge using a microsyringe (Henseki, Switzerland). MFSD2A‐OE group received 2 μL of synthetic virus rAAV‐tie2‐MFSD2A‐2a‐EGFP‐3xmir122a‐WPRE, MFSD2A‐NC group received 2 μL of control virus. CTSS‐KD group received 2 μL of synthetic virus rAAV‐U6‐shRNA (CTSS)‐CMV‐EGFP‐pA, CTSS‐NC group received 2 μL of control virus. Mice were observed for general condition post‐injection; those with cataract formation were excluded from the experiment. Samples were collected 4 weeks after intravitreal injection for subsequent analysis.

### 2.3 HE and Immunohistochemical Staining

Eyeball specimens in paraformaldehyde were embedded in paraffin, cut into 4 μm sections and stained with hematoxylin eosin. Sections were dewaxed with xylene, rehydrated with gradient ethanol, blocked with 10% goat serum for 30 min, and incubated with anti‐TNF‐α (Abcam, ab307164, 1:200), anti‐IL‐1β (Abcam, ab283818, 1:200), anti‐TGF‐β (Abcam, ab215715, 1:200), and anti‐IL‐10 (HUABIO, HA722032, 1:400) primary antibody at 4°C overnight, respectively. After washing with PBS, the sections were incubated with secondary antibody for 50 min, washed with PBS, treated with 3,3′‐diaminobenzidine for 3 min, and counterstained with hematoxylin. Images were taken at ×400 original magnification (Nikon, Japan) and analyzed with ImageJ.

### 2.4 Immunofluorescence Staining

Sections were dewaxed with xylene, rehydration with gradient ethanol, blocked with 10% goat serum for 30 min, incubated with anti‐CD31 (Abcam, ab281583, 1:1000), anti‐MFSD2A (Proteintech, 30555‐1‐AP, 1:200), anti‐Iba‐1 (Abcam, ab178847, 1:1000), anti‐CTSS (Santa, SC‐74429, 1:100), anti‐CD86 (CST, 19589, 1:100), anti‐CD206 (Abcam, ab64693, 1:500) primary antibody at 4°C overnight, respectively. After washing with TBST, sections were incubated with secondary antibody (Donkey anti‐Mouse IgG or Donkey anti‐Rabbit IgG, ThermoFisher, 1:400) for 50 min. After TBST washing, added 50–100 μL of DAPI working solution (Solarbio, 1:500) to each slice, stained the nucleus in dark for 5 min, and rinsed with PBS. After drying the slices, sealed them with fluorescent sealing agent and stored at 4°C in dark. Images were taken at ×400 original magnification (Nikon, Japan), analyzed with ImageJ.

### 2.5 Transmission Electron Microscopy

Mice were anesthetized with isoflurane and euthanized. Eyes were quickly removed using ophthalmic forceps, after eyeball surface and intravitreous injection with 2.5% glutaraldehyde (Sinopharm Chemical Reagent, Shanghai); eyeballs were fixed in 2.5% glutaraldehyde for 24 h, followed by fixation in 1% osmium tetroxide (Alfa Aesar, Shanghai). After dehydration in a graded ethanol series and embedding in epoxy resin (Shresin, Shanghai), eye specimens were sectioned using an ultramicrotome (Leica, Germany), stained with lead citrate (Sinopharm Chemical Reagent, Shanghai), observed, and photographed under a transmission electron microscope (Hitachi, H‐7700, Japan).

### 2.6 Retinal Leakage Detection

FITC‐dextran (Sigma, FD2000S) was dissolved in physiological saline to prepare a 15 mg/mL solution. After anesthetizing mice, microvascular leakage in whole‐mounted retinas was determined by fluorescein angiography as previously described [13].

### 2.7 Single‐Cell RNA Sequencing (scRNA‐Seq) Analysis

Retinal single‐cell suspensions were prepared by pooling left eye retinas from three mice per biological replicate (*n* = 3 replicates per group). Briefly, dissected retinas were enzymatically digested, filtered through a 40 μm strainer, and washed. Red blood cells were lysed, and cell viability was assessed by trypan blue exclusion (≥ 85% viability required). Single‐cell libraries were constructed using the Singleron Matrix system and GEXSCOPE kits according to the manufacturer's protocol [14]. Sequencing was performed on an Illumina NovaSeq 6000 platform (150 bp paired‐end).

Raw reads were processed using CeleScope v1.12.0 to generate gene expression matrices. Reads were aligned to the mm10 genome with STAR v2.6.1a [15], and gene/UMI counts were quantified with featureCounts v2.0.1 [16]. Quality control, normalization, and clustering were performed using Scanpy [17]. Cells were filtered if they had < 200 genes, were in the top 2% for gene/UMI counts, or had > 30% mitochondrial content. Genes detected in < 5 cells were removed. After normalization and log‐transformation, the top 2000 variable genes were selected for PCA. The top 20 principal components were used for Louvain clustering (resolution = 1.2) and visualization with UMAP.

Differentially expressed genes (DEGs) were identified using the Seurat FindMarkers function (Wilcoxon test), requiring expression in > 10% of cells and |logFC| > 0.25. Cell types were annotated based on canonical marker expression and the SynEcoSys database. Doublets were manually removed. Pathway enrichment analysis was performed using GO and KEGG via clusterProfiler [18]. Cell–cell interactions were inferred with CellPhoneDB v2.1.0 [19], using 1000 permutations and a significance threshold of *p* < 0.05 with average log expression > 0.1.

### 2.8 Unmarked 3D Tomographic Scanning Microscopic Imaging

Primary human retinal microvascular endothelial cells (HRMECs, iCell, Shanghai) and human microglial cell line (HMC3, Procell, Wuhan) were separately seeded into 35 mm μ‐culture dishes and cultured in endothelial or microglial culture media. Normal glucose group (NG, 5.5 mmol/L glucose) received no additional interventions. High glucose group (HG) was intervened with 55 mmol/L glucose + LPS (1 μg/mL) for 24 h. A 2D co‐culture system of microglia and endothelial cells was prepared in the culture dishes, with approximately 20,000 cells of each cell type [20]. After 24 h of intervention, suitable cell densities were observed and the culture dishes were transferred to the operation platform of the 3D holographic tomographic scanning microscope. Holographic images were obtained using a 3D cell exploration microscope equipped with a 60‐fold magnification (*λ* = 520 nm, sample exposure 0.2 mW/mm^2^) and a 30 μm depth of field.

### 2.9 Cell Culture

HRMECs and HMC3 were cultured in a CO_2_ incubator at 37°C with 5% CO_2_. HRMECs were cultured in primary endothelial cell basal medium (iCell, Shanghai), while HMC3 were cultured in MEM (containing NEAA, Procell, Wuhan) supplemented with 10% FBS and 1% P/S.

### 2.10 Transfection of MFSD2A Overexpression Plasmid Into HRMECs

Well‐maintained HRMECs were passaged and divided into NG, HG, NG + MFSD2A‐OE, and HG + MFSD2A‐OE groups. NG + MFSD2A‐OE group and HG + MFSD2A‐OE group were intervened with MFSD2A overexpression plasmid (Zixibio, Beijing) and lip3000 pre‐mixed transfection reagent (Lipofectamine RNAiMAX Reagent, Invitrogen) for 6 h. After transfection, NG group and NG + MFSD2A‐OE group continued to be intervened with normal glucose medium (5.5 mmol/L) for 24 h, while HG and HG + MFSD2A‐OE groups were switched to 55 mmol/L glucose + 1 μg/mL LPS for 24 h. Cells were collected and stored at −80°C.

### 2.11 Transfection of CTSS siRNA Into HMC3

Well‐maintained HMC3 were divided into NG, HG, NG + siCTSS, and HG + siCTSS groups. NG + siCTSS and HG + siCTSS groups were intervened with CTSS siRNA (Zixibio, Beijing) and lip3000 pre‐mixed transfection reagent (LipofectamineTM RNAiMAX Reagent, Invitrogen) for 6 h. After transfection, NG and NG + siCTSS groups continued to be intervened with normal glucose medium (5.5 mmol/L) for 24 h, while HG and HG + siCTSS groups were switched to 55 mmol/L glucose + 1 μg/mL LPS for 24 h. Cells were collected and stored at −80°C.

### 2.12 Exogenous CTSS Intervention in HRMECs

Established a high glucose intervention HRMECs model with 55 mmol/L glucose +1 μg/mL LPS for 24 h. Similarly, the HRMECs PAR2 knockdown model was established with PAR2 siRNA. 6 h after transfection of PAR2 siRNA into HRMECs, exogenous addition of different concentration of CTSS (0.5, 1.5, and 5 ng/mL) for intervention on HRMECs. After transfection, the groups were intervened with high glucose medium (55 mmol/L glucose +1 μg/mL LPS) for 24 h. Cells were collected and stored at −80°C.

### 2.13 Transwell Co‐Culture System

Established a high glucose intervention microglia‐endothelial cell transwell model, with microglia in the upper inserts (COStar, 0.4 μm pore size, US) and endothelial cells in the well plate. HRMECs PAR2 knockdown and HMC3 CTSS knockdown models were established with PAR2 siRNA and CTSS siRNA, respectively. 6 h after transfection of PAR2 siRNA and CTSS siRNA, cells were intervened with either normal glucose or high glucose for 24 h. Cells were collected and stored at −80°C.

### 2.14 CCK‐8 Assay for Cell Viability

Under dark conditions, reagents were added according to instructions of CCK‐8 assay kit (Beyotime, Shanghai). The absorbance value at a wavelength of 450 nm was measured using a microplate reader (Biobase, Shandong) as an indicator of cell viability assessment.

### 2.15 qRT‐PCR

Total RNA from the cell lines was extracted with TRIzol reagent. cDNA was synthesized using the SuperScript III reverse transcriptase kit (Invitrogen, 11752050). Relative RNA levels were determined by PCR amplification performed on a real‐time PCR system (Applied biosystems, USA, StepOne Software) with the SYBR Green reagent using the 2^−∆∆Ct^ method. β‐actin was employed as an internal control for quantifications. The sequences of the gene‐specific primers are listed in Table 1.

**TABLE 1:** The primers used for qRT‐PCR.

Gene	Primer sequence
MFSD2A‐F	5′‐CCTTGTTTCCAGGACCTCAA‐3′
MFSD2A‐R	5′‐GAAGTAGGCGATTGGCTCAG‐3′
CAV‐1‐F	5′‐TCTCTCTTTCCTGCACATCTGG‐3′
CAV‐1‐R	5′‐AACAGCTTCAAAGAGTGGGTCA‐3′
Occludin‐F	5′‐CCCCATCTGACTATGTGGAA‐3′
Occludin‐R	5′‐TAGGAAGACTCTGGATAAAACCG‐3′
Claudin‐5‐F	5′‐TGACCGCCTTCCTGGACCACAAC‐3′
Claudin‐5‐R	5′‐CTGCACCACGCACGACATCCACA‐3′
ZO‐1‐F	5′‐TTCCGTGTTGTGGATACCTTG‐3′
ZO‐1‐R	5′‐ATGATGCCTCGTTCTACCTC‐3′
CTSS‐F	5′‐TGACAACGGCTTTCCAGTACA‐3′
CTSS‐R	5′‐GGCAGCACGATATTTTGAGTCAT‐3′
PAR2‐F	5′‐TCCTCACAGCCTCTGCCTAT‐3′
PAR2‐R	5′‐AGACAAAGGGGTCGATGCAG‐3′
β‐Actin‐F	5′‐TCCTCCTGAGCGCAAGTACTCC‐3′
β‐Actin‐R	5′‐CATACTCCTGCTTGCTGATCCAC‐3′

### 2.16 Western Blot

The samples were washed with cold PBS and homogenized in 1 × RIPA buffer (20 mM Tris, pH = 7.5, 150 mM NaCl, 1% Triton X‐100, sodium pyrophosphate, β‐glycerophosphate, EDTA, Na3VO4 and leupeptin, Beyotime, P0013). The samples were subjected to SDS‐PAGE and transferred to PVDF membrane (Millipore, USA). The membrane was blocked with 5% BSA and then incubated with primary antibodies of anti‐MFSD2A (Proteintech, 67965‐1‐Ig, 1:10,000), anti‐CAV‐1 (Affinity, AF6386, 1:1000), anti‐CTSS (Abmart, T58513S, 1:1000), anti‐PAR2 (Abcam, ab180953, 1:5000), anti‐ZO‐1 (Affinity, AF5145, 1:1000), anti‐Occludin (Affinity, DF7504, 1:2000), and anti‐Claudin‐5 (Affinity, AF5216, 1:1000) antibody at 4°C overnight. Anti‐β‐actin (Proteintech, 66009‐1‐Ig, 1:50,000) was served as control. Then the blots were incubated with an HRP‐conjugated secondary antibody Goat Anti‐Rabbit IgG (Abcam, ab205718, 1:20,000) or Goat Anti‐Mouse IgG (Abcam, ab205719, 1:10,000) for 1 h at room temperature. The immunoreactive bands were visualized by chemiluminescence imager (Clinx, RT‐YQSB‐0556, Shanghai). For quantification, the protein band intensities of the western blot images were measured with ImageJ software (National Institutes of Health, Bethesda, MD).

### 2.17 Retro‐Orbital Blood Collection and ELISA Detection

Blood samples were collected from the retro‐orbital plexus/vein under isoflurane anesthesia using heparinized capillary tubes, as previously described with modifications [21]. The collected blood was centrifuged promptly (4°C, 3000*g* for 15 min). The resulting plasma supernatant was carefully separated and stored at −80°C until analysis. To aid recovery of blood volume and prevent hypovolemic shock, mice could receive a subcutaneous or intraperitoneal injection of 0.2–0.5 mL sterile saline solution post‐procedure. Plasma insulin levels were quantified using a commercially available enzyme‐linked immunosorbent assay kit (ELISA) according to the manufacturer's instructions (Proteintech, KE10089). Collected cell culture supernatant. Detected the concentration of cytokines according to CTSS (MDL, MD42561, Hebei), IL‐1β (MDL, MD56739, Hebei), TNF‐α (MDL, MD120512, Hebei), IL‐10 (MDL, MD132647, Hebei), and TGF‐β (MDL, MD132845, Hebei) ELISA test kit instructions.

### 2.18 Flow Cytometry

Digested cells with pancreatic enzymes (Gibco, 25200‐072), after washing with PBS, cells were resuspended with culture medium, and adjusted cell concentration to 1 × 10^6^ cells/100 μL. Added FITC‐anti‐human CD16 (BioLegend, 302006), PE‐anti‐human CD68 (BioLegend, 333808), APC‐anti‐human CD206 (BioLegend, 321110) according to the instructions, incubated at room temperature in the dark for 30 min, and set up a blank control group (without any antibodies). Centrifuged and discarded the supernatant. After PBS washing, resuspended the cells, detected and analyzed by flow cytometry (BEAM DIAG, BeamCyte‐1026).

### 2.19 Transwell Transcytosis Assay

Endothelial cells were seeded on the upper inserts of transwell and cultured to confluence. After transfection of MFSD2A overexpression plasmid and HG, 2.5 mg/mL HRP (Solarbio, P8020, Beijing) was added to the upper inserts of transwell and incubated at 37°C for 20 min. Then the inserts were washed with cold PBS for several times to remove the free extracellular HRP. The medium was provided to both upper and lower inserts with 60 min incubation at 37°C. The lower well plate medium was collected, and the concentration of HRP was measured according to the instructions of the QuantaBlu Fluorogenic Peroxidase Substrate Kit (Thermo Fisher Scientific, 15169).

### 2.20 Statistical Analysis

Processed experimental data using GraphPad Prism 8.0.1. Data were represented as mean ± SD. Double tailed Student *t*‐test for comparison between two groups, statistical differences among three or more groups were evaluated using one‐way ANOVA. *p* < 0.05 was considered significance. *p < 0.05, **p < 0.01, ***p < 0.001.

## 3 Results

### 3.1 Single‐Cell Transcriptome Atlas

Baseline measurements showed no intergroup differences in body weight, fasting glucose, or serum insulin. Post‐STZ induction, sustained hyperglycemia, progressive weight loss, and declining insulin levels confirmed successful diabetic modeling (all *p* < 0.001 vs. Control). In DR retinas, marked basement membrane thickening (172.27 ± 41.99 vs. 81.07 ± 6.27 nm, *p* = 0.021) and severe vascular leakage (14.86% ± 0.76% vs. 3.87% ± 0.88%, *p* < 0.001) were observed, alongside vacuolar degeneration and disrupted cellular architecture, confirming early diabetic retinopathy features (Supporting Information 1).

scRNA‐seq enables high‐resolution characterization of cell type‐specific transcriptional heterogeneity and intercellular signaling networks [22]. Retinal single cell suspension was prepared from Control and DR mice retina, respectively (*n* = 3 for biological replicate). After rigorous quality control and batch effect correction, integrated analysis revealed 14,207 cells from Control and 13,607 cells from DR retinas. Computational clustering via CellID algorithm identified eight distinct retinal cell populations (Figure 1A–D): Müller cells, Amacrine cells, Bipolar cells, Retinal Cone cells, Retinal Rod cells, Endothelial cells (ECs), Mural cells, and Mononuclear Phagocytes (MPs). MPs exhibited strong enrichment of microglial markers (P2ry12, Cx3cr1, C1qc, Figure 1E), and microglia comprise > 90% of retinal immune cells [23]. We also proved the negligible expression change between DR and Control retinas through immunohistochemical staining (CD3, CD20, CD68, CD11b, MPO, Supporting Information 2), then we analyzed MPs as microglia in subsequent analysis.

**FIGURE 1:**
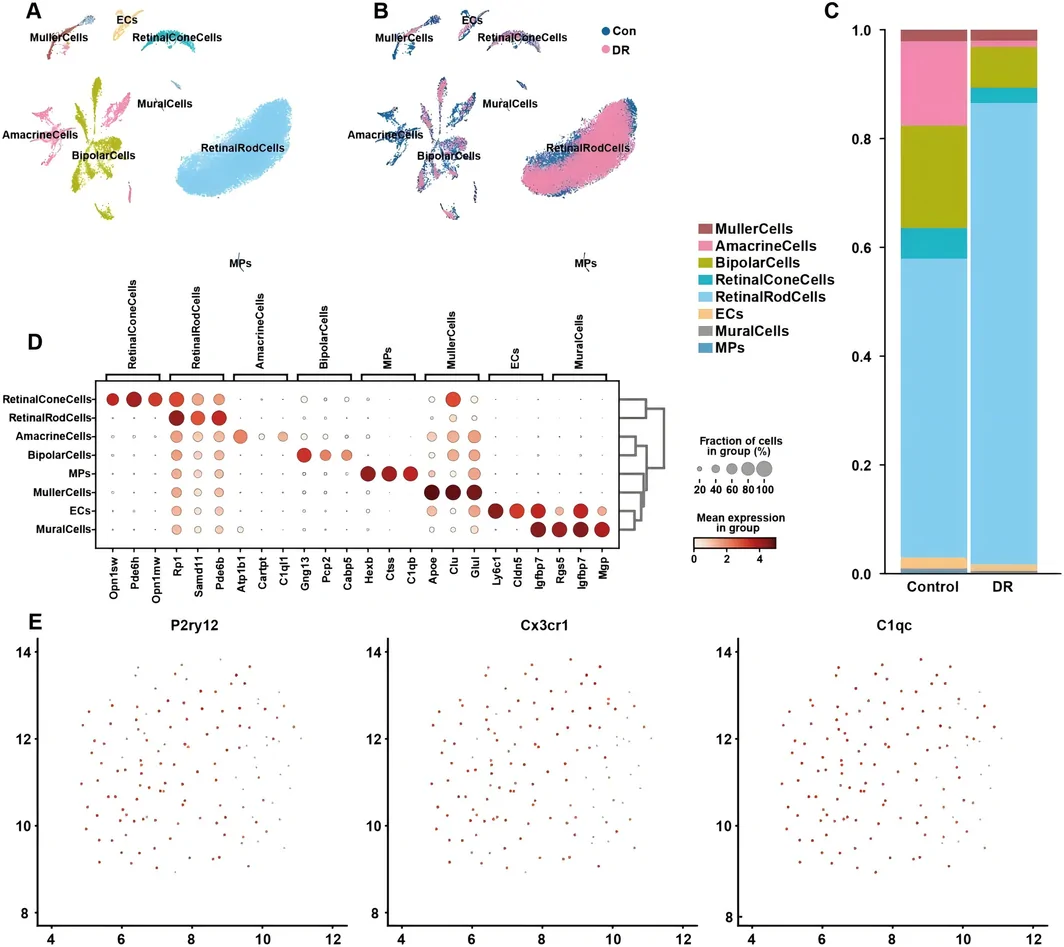
Single‐cell transcriptome atlas. (A) UMAP plot with annotations of major retinal cell types. (B) Different color distinguish distribution of retinal cell types between Control and DR group, with blue for Control and pink for DR group. (C) Histogram of proportion of various retinal cell types between Control and DR group. (D) Bubble plot of top 3 markers in each retinal cell type. (E) Expression of microglial specific markers (P2ry12, Cx3cr1, C1qc) in MPs. Bright yellow dots represent gene positive cells, gray dots represent gene negative cells.

Microglial decline (Control vs. DR, 0.56% vs. 0.15%) may reflect a compensatory anti‐inflammatory mechanism during early DR pathogenesis, where resident microglia limit neuroinflammatory cascades through population control [24]. Concurrent neuronal loss (amacrine/bipolar cells) correlates with emerging scotopic dysfunction [23], while endothelial depletion suggests hyperglycemia‐induced vascular apoptosis [25]. The rod dominance (84.86%) implies metabolic reprogramming favoring phototransduction machinery preservation [26]. The above evidence suggests that retinal metabolic disorder in early diabetes may induce immune inflammation imbalance and aggravate neurovascular injury.

### 3.2 Diabetes‐Induced Metabolic Dysregulation Triggers CTSS Expression in Activated Retinal Microglia

ScRNA‐seq characterized the main function and change of microglia in the retina under diabetes (Supporting Information 3), indicating that microglia maintain inflammatory homeostasis through early reactivity, high migratory, and phagocytic activity. Retinal immunofluorescence staining validated microglia predominantly localized in the retinal ganglion cell (RGC) layer, inner plexiform layer (IPL), and outer plexiform layer (OPL), exhibiting a ramified morphology with elongated, branching processes (yellow arrows in Figure 2B) in the Control retina. While microglia transformed into amoeboid‐like cells characterized by enlarged somata and truncated processes in the DR retina (yellow arrows in Figure 2B). Immunohistochemical staining under brightfield microscopy further demonstrated microglial morphology and distribution changes (Figure 2C), indicating microglial activation, morphological remodeling, and redistribution in early diabetes.

**FIGURE 2:**
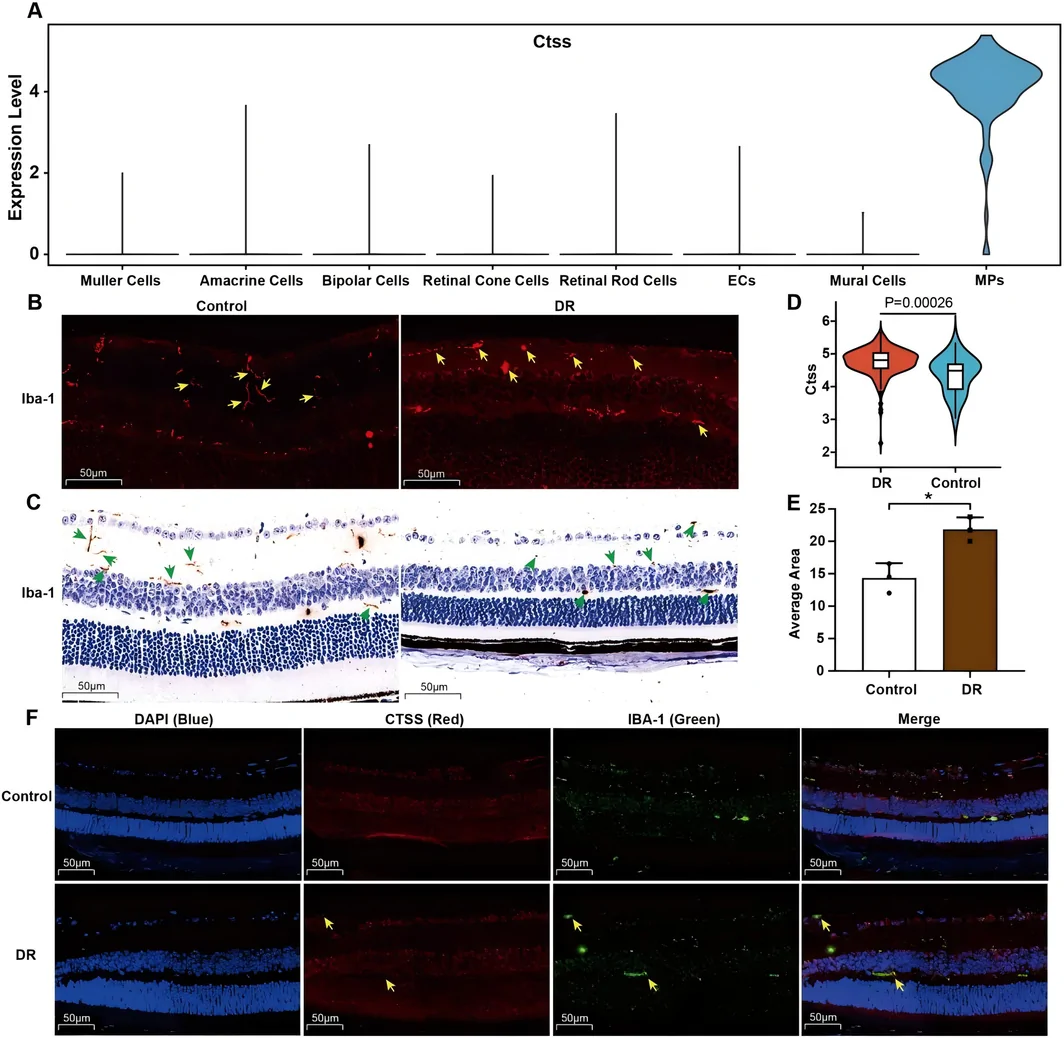
Diabetes‐induced metabolic dysregulation triggers CTSS expression in activated retinal microglia. (A) Violin plot of CTSS expression in various retinal cell types based on scRNA‐seq. (B) Immunofluorescence staining of retinal microglia (Iba‐1+), the yellow arrows indicate microglial morphological changes. (C) Immunohistochemical staining of retinal microglia, the green arrows indicate microglial morphological changes. (D) Expression of retinal microglial Ctss gene between DR and Control group based on retinal scRNA‐seq. (E) Statistical analysis based on the average double positive (IBA‐1+CTSS+) fluorescence area (*n* = 3). (F) Immunofluorescence staining of microglia (Iba‐1+) and CTSS. The yellow arrows indicate the double positive (IBA‐1+CTSS+) area. Data represents as mean ± SD. ns, no significance. *p < 0.05.

ScRNA‐seq analysis demonstrated predominant CTSS expression in retinal microglia (Figure 2A). ScRNA‐seq data in our previous study [27] proved diabetes significantly elevated CTSS mRNA expression in retinal microglia (log_2_FC = 0.4952, *p* = 0.0003 vs. Control, Figure 2D and Supporting Information 4), suggesting that retinal metabolic dysregulation may exert a pronounced effect on CTSS expression. Immunofluorescence quantification validated diabetes‐induced CTSS upregulation in activated retinal microglia (*p* = 0.0119, Figure 2E,F).

### 3.3 CTSS Drives Pro‐Inflammatory Polarization of Microglia in Diabetic Retinopathy

Based on our previous scRNA‐seq data [27], we characterized the main function of retinal CTSS using Gene Set Enrichment Analysis (GSEA), histopathological staining, and western blot, validated successful establishment of retinal microglial CTSS knockdown model in diabetic mice (Supporting Information 5). Further histopathological staining preliminarily demonstrates that diabetes‐activated microglia amplified retinal inflammatory cascades through CTSS‐dependent mechanisms, knocking down retinal microglial CTSS inhibited polarization of microglial pro‐inflammatory phenotype and expression of inflammatory factors (TNF‐α and IL‐1β, Supporting Information 6 and 7).

We first validated the knockdown efficiency of CTSS under NG and HG (Figure 3A–DF), demonstrating the successful establishment of the CTSS knockdown model. CCK‐8 assays confirmed that HG did not affect microglial viability (*p* = 0.9137 vs. NG, Figure 3E). HG increased M1 phenotype proportion (*p* < 0.001 vs. NG, Figure 3G,I), upregulated pro‐inflammatory cytokines IL‐1β (*p* < 0.001 vs. NG) and TNF‐α (*p* < 0.001 vs. NG, Figure 3K,L), reduced anti‐inflammatory cytokines IL‐10 (*p* < 0.001 vs. NG) and TGF‐β (*p* < 0.001 vs. NG, Figure 3M,N), while not affecting M2 phenotype proportion (*p* = 0.3692 vs. NG, Figure 3H,J). Notably, HG + siCTSS reduced M1 polarization (*p* = 0.0009 vs. HG, Figure 3G,I), suppressed IL‐1β (*p* < 0.001 vs. HG) and TNF‐α expression (*p* < 0.001 vs. HG, Figure 3K,L), partially restored IL‐10 (*p* < 0.001 vs. HG) and TGF‐β expression (*p* < 0.001 vs. HG, Figure 3M,N). These findings demonstrate that HG‐induced CTSS overexpression drives microglial activation toward a pro‐inflammatory phenotype, disrupts cytokine equilibrium, and establishes a pro‐inflammatory microenvironment; knocking down retinal microglial CTSS inhibited polarization of the microglial pro‐inflammatory phenotype and expression of inflammatory cytokines.

**FIGURE 3:**
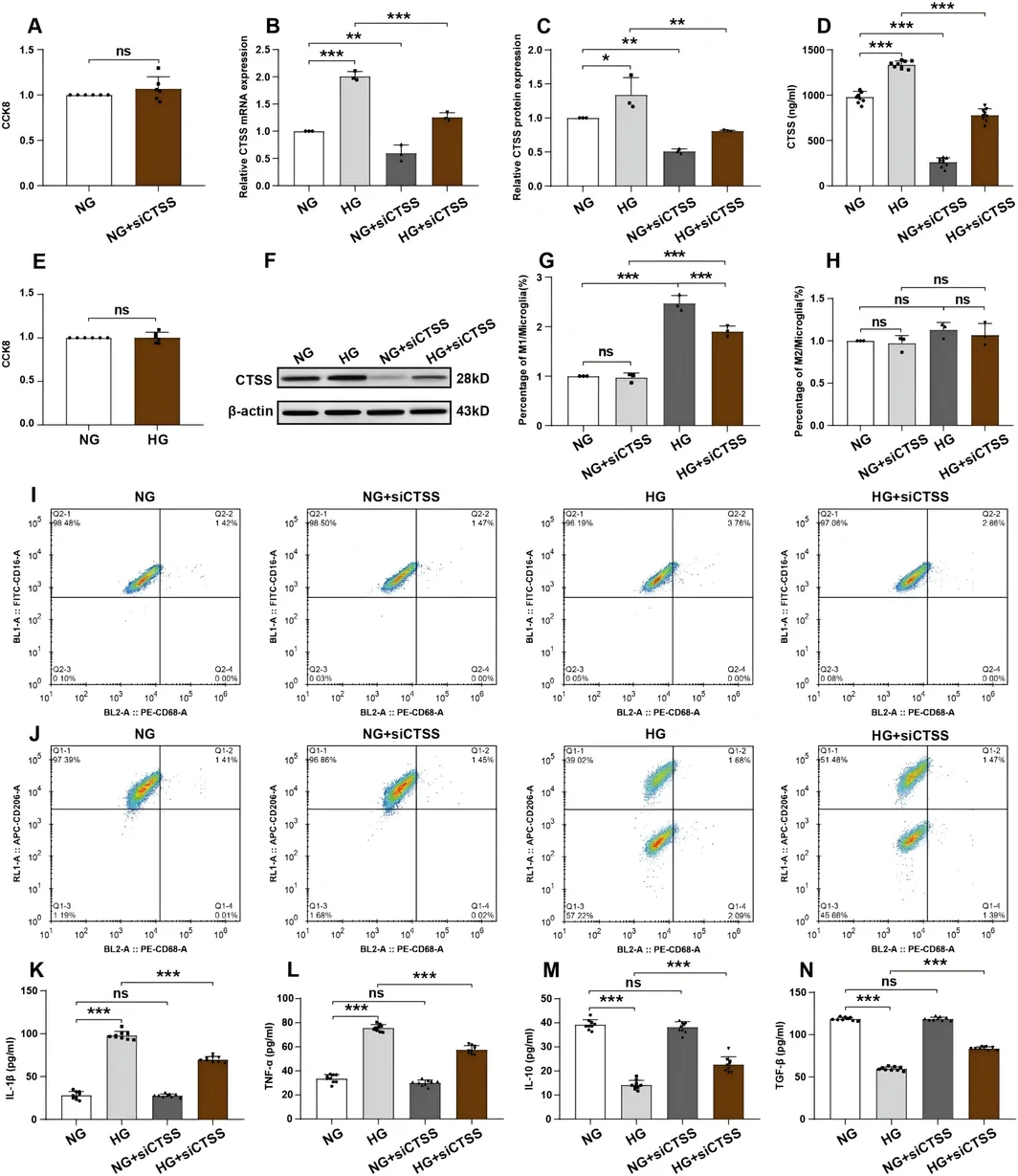
CTSS drives pro‐inflammatory polarization of microglia in diabetic retinopathy. (A) CCK8 results of HMC3 viability (*n* = 6). (B) Relative expression of HMC3 CTSS mRNA (*n* = 3). (C) Relative expression of HMC3 CTSS protein based on western blot (*n* = 3). (D) CTSS concentration in the cell culture supernatant of HMC3 (*n* = 9). (E) CCK8 results of HMC3 viability (*n* = 6). (F) Protein bands of HMC3 CTSS. (G) Statistical analysis of flow cytometry results of M1 (*n* = 3). (H) Statistical analysis of flow cytometry results of M2 (*n* = 3). (I) Flow cytometry results of M1 (CD16+CD68+). (J) Flow cytometry results of M2 (CD16+CD206+). IL‐1β (K), TNF‐α (L), IL‐10 (M) and TGF‐β (N) concentration in the cell culture supernatant of HMC3 (*n* = 9). Data represents as mean ± SD. ns, no significance. *p < 0.05, **p < 0.01, ***p < 0.001.

### 3.4 MFSD2A Downregulated in Retinal Endothelial Cells in Early Diabetes

As the primary cellular constituent of inner blood‐retinal barrier (iBRB), endothelial cells directly interface with blood components and serve as frontline responders to systemic metabolic disturbances [22]. We characterized the main function and change of retinal endothelial cells using scRNA‐seq (Supporting Information 8), indicating that endothelial cells participate in the regulation of vasculature development by maintaining barrier function. Further, scRNA‐seq analysis revealed predominant Mfsd2a expression in retinal endothelial cells (Figure 4A) and a reduction in the DR group (*p* = 0.01 vs. Control, Figure 4B,C). Immunohistochemistry and immunofluorescence quantification confirmed MFSD2A downregulation in the whole retina (*p* = 0.0002 vs. Control, Figure 4D,E) and in retinal endothelial cells, respectively (*p* = 0.0040 vs. Control, Figure 4F,G) in the DR group. These findings show that MFSD2A is constitutively expressed in retinal endothelial cells, and diabetes‐induced MFSD2A suppression occurs during early diabetes.

**FIGURE 4:**
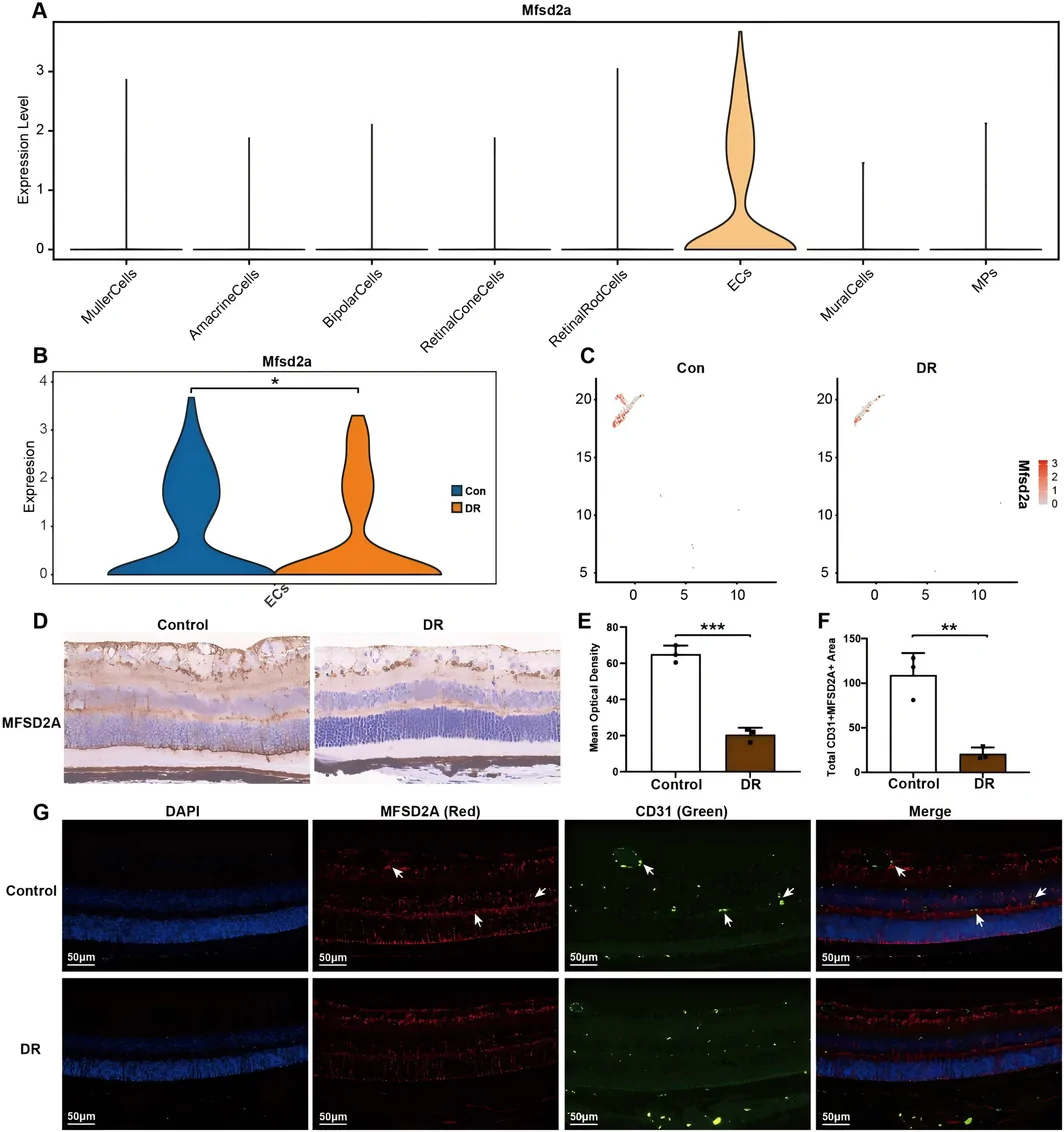
MFSD2A downregulated in retinal endothelial cells in early diabetes. (A) Violin plot of Mfsd2a expression in various retinal cell types based on scRNA‐seq. (B) Differential expression of endothelial Mfsd2a between Control and DR group (violin plot). (C) Differential expression of endothelial Mfsd2a between Control and DR group (feature plot). Bright yellow dots represent gene expression positive cells, the redder the color, the higher the gene expression level. (D) Retinal immunohistochemical staining images. (E) Statistical analysis based on retinal immunohistochemical staining (*n* = 3). (F) Statistical analysis based on retinal immunofluorescence staining, the data is represented as the total area of CD31+MFSD2A+ double positive area (*n* = 3). (G) Immunofluorescence staining of retinal endothelial cells (CD31+) and MFSD2A. The white arrows represent the CD31+MFSD2A+ double positive area. Data represents as mean ± SD. *p < 0.05, **p < 0.01, ***p < 0.001.

### 3.5 Endothelial MFSD2A Overexpression Ameliorates Diabetic Vascular Leakage by Suppressing Transcytosis

We established retinal endothelial MFSD2A overexpression model in diabetic mice and validated by histopathological staining and HRMEC western blot (Supporting Information 9). TEM revealed retinal structure of Control group remained compact, featuring intact mitochondria, thin vascular BM, densely stained nuclei, uniform cytoplasm, and minimal transcytosis (yellow arrows in Figure 5A). DR group exhibited structural deterioration, including mitochondrial vacuolation, BM thickening (*p* = 0.0224 vs. Control), cytoplasm vacuolar degeneration, increased transcytosis (*p* = 0.0067 vs. Control, yellow arrows in Figure 5A). MFSD2A‐NC mirrored DR pathology (BM thickness: *p* = 0.9894 vs. DR, transcytosis: *p* = 0.9475 vs. DR). MFSD2A‐OE partially restored BM thickness (*p* = 0.0159 vs. DR, Figure 5C), reduced transcytosis (*p* = 0.008 vs. DR, Figure 5D) and improved endothelial cytoarchitecture.

**FIGURE 5:**
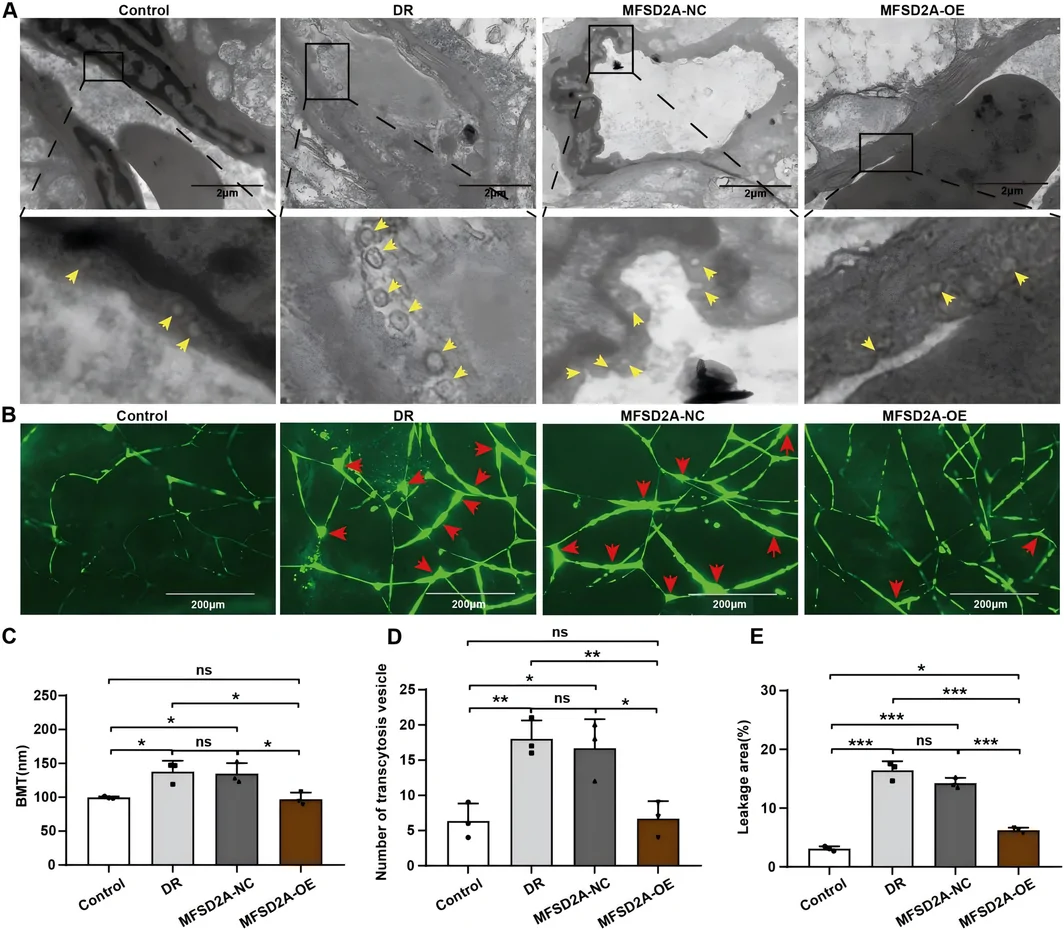
Endothelial MFSD2A overexpression ameliorates diabetic vascular leakage by suppressing transcytosis. (A) Retinal TEM images, the yellow arrows indicate transcytosis vesicles. (B) Vascular permeability results detected by FITC‐dextran, the red arrows indicate fluorescence leakage. (C) Statistical analysis of vascular BMT. BMT presents as the average of five points in each retina (*n* = 3). (D) Statistical analysis based on number of transcytosis vesicles in each field of TEM (×5k). (E) Statistical analysis of proportion of fluorescein leakage area. Select one image from each of the retinal upper, lower, left, and right quadrants, data presents as mean of proportion of retinal leakage area of the four quadrants (*n* = 3). Data represents as mean ± SD. ns, no significance. *p < 0.05, **p < 0.01, ***p < 0.001.

We further performed FITC‐dextran to evaluate retinal vascular permeability, demonstrating that preserved vascular integrity in Control group, with intact vascular permeability restricting fluorescence to the intravascular compartment (Figure 5B). DR group exhibited pronounced perivascular fluorescence clusters (red arrows in Figure 5B), reflecting vascular leakage (*p* < 0.001 vs. Control). MFSD2A‐OE (*p* < 0.001 vs. DR) rather than MFSD2A‐NC (*p* = 0.0877 vs. DR) group markedly reduced fluorescence extravasation, though residual leakage persisted (*p* = 0.0152 for MFSD2A‐OE vs. Control, Figure 5B,E). Collectively, these results demonstrate that diabetes‐induced MFSD2A downregulation drives retinal vascular hyperpermeability, while targeted endothelial MFSD2A overexpression partially restores barrier function through inhibiting transcytosis.

### 3.6 MFSD2A Regulates Vascular Permeability via Transcytosis‐Dependent Mechanism Independent of Tight Junction

By establishing HRMECs Mfsd2a overexpression model based on HG, HG suppressed endothelial Mfsd2a expression (mRNA, *p* = 0.0278 vs. NG, protein, *p* = 0.0040 vs. NG), horseradish peroxidase (HRP) permeability assays (excluding paracellular leakage [8]) revealed HG increased HRP leakage by 51% (*p* < 0.001 vs. NG, Figure 6D). MFSD2A‐OE under HG restored MFSD2A expression (mRNA, *p* < 0.001 vs. HG, protein, *p* = 0.0002 vs. HG, Figure 6A–C), MFSD2A‐OE reduced HG‐induced leakage by 50% (*p* < 0.001 vs. HG, Figure 6D). These findings confirm that MFSD2A overexpression counteracts diabetes‐driven vascular hyperpermeability through inhibiting transcytosis.

**FIGURE 6:**
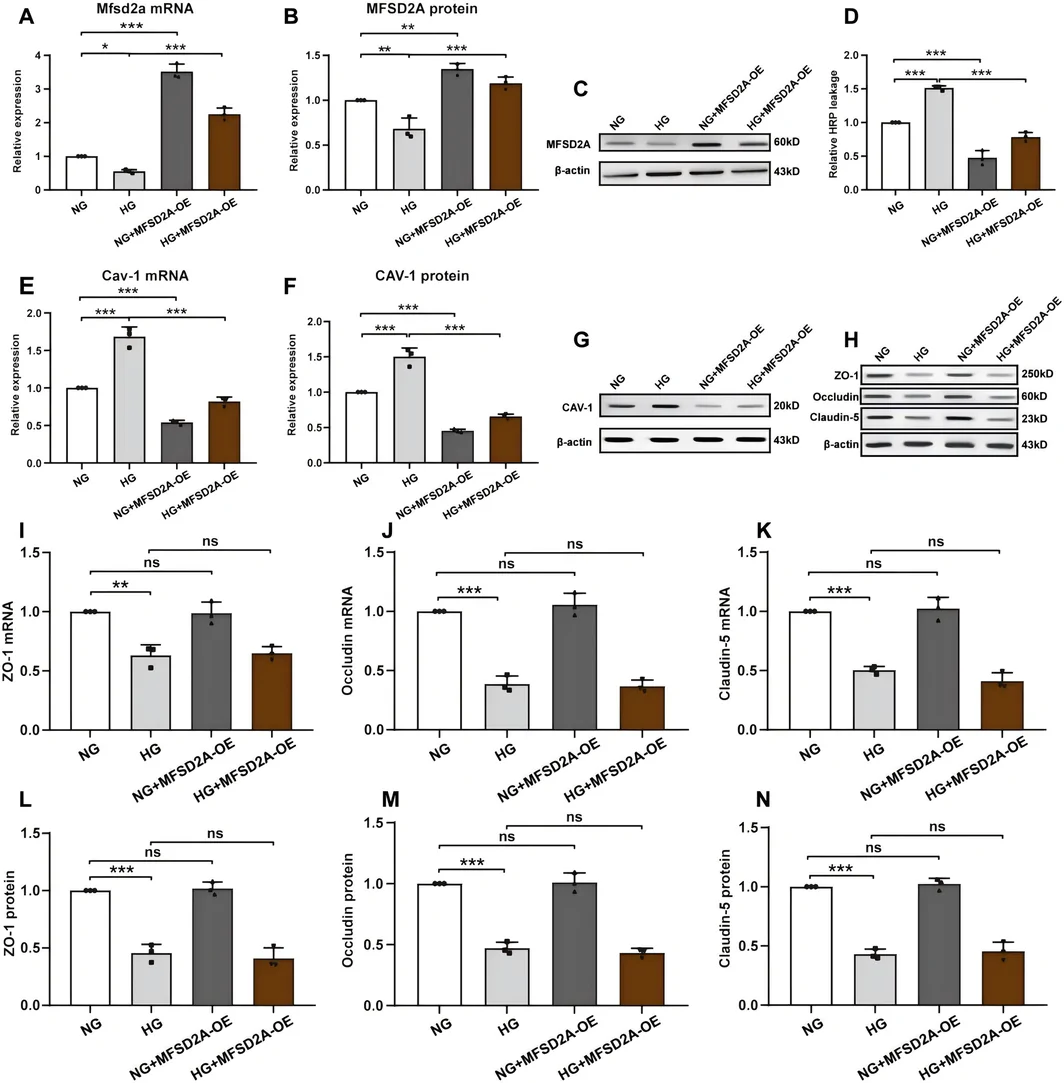
MFSD2A regulates vascular permeability via transcytosis‐dependent mechanism independent of tight junction. Relative expression of HRMECs Mfsd2a mRNA (A) and protein (B) (*n* = 3). (C) Protein bands of HRMECs MFSD2A. (D) Relative HRP leakage (*n* = 3). Relative expression of HRMECs Cav‐1 mRNA (E) and protein (F) (*n* = 3). (G) Protein bands of HRMECs CAV‐1. (H) Protein bands of ZO‐1, Occludin and Claudin‐5. Relative expression of ZO‐1 mRNA (I), Occludin mRNA (J) and Claudin‐5 mRNA (K) (*n* = 3). Relative expression of ZO‐1 protein (L), OCCLUDIN protein (M) and CLAUDIN‐5 protein (N) (*n* = 3). Data represent as mean ± SD. ns, no significance. *p < 0.05, **p < 0.01, ***p < 0.001.

CAV‐dependent transcytosis critically impacts endothelial transcytosis, signal transduction, and regulates vascular permeability [28]. We confirmed HG upregulated CAV‐1 expression (mRNA: *p* < 0.001 vs. NG, protein: *p* < 0.001 vs. NG), MFSD2A‐OE reversed HG‐induced CAV‐1 elevation (mRNA: *p* < 0.001 vs. HG, protein: *p* < 0.001 vs. HG, Figure 6E–G). Consistent with our study, Wang et al. [8] demonstrated that MFSD2A actively participates in caveolae‐mediated transcytosis in endothelial cells, thereby regulating transcytosis and influencing the integrity of the iBRB. Confirming that MFSD2A‐mediated regulation of transcytosis is CAV‐1‐dependent. Furthermore, we also investigate whether MFSD2A‐mediated transcytosis interacts with tight junction proteins, and confirming that MFSD2A regulated transcytosis function independently of canonical tight junction protein dynamics (Figure 6I–N), representing a distinct pathway for vascular permeability regulation in DR.

### 3.7 CTSS‐PAR2 Mediates Microglial‐Endothelial Crosstalk and Regulates Endothelial MFSD2A Expression

We characterized microglia‐endothelial crosstalk using CellPhoneDB‐based ligand–receptor interaction analysis, holographic 3D tomography imaging, and a 2D co‐culture model, demonstrated symbiotic microglia‐endothelial interactions in NG, while dynamic process extension‐retraction cycles presented in HG: first, microglial processes actively contact endothelial cells, then rapid retraction post‐endothelial apoptosis induction (Supporting Information 10 and video materials as Supporting Information 11A,B). Indicating that hyperglycemia disrupts physiological microglia‐endothelial communication, promoting cytotoxic interactions and driving vascular injury.

CTSS activates protease‐activated receptor 2 (PAR2) through proteolytic cleavage of its extracellular N‐terminal domain [29]. We knocked down PAR2 with siRNA transfection and validated the knockdown efficiency of PAR2 under NG and HG (Figure 7A–C). Exogenous CTSS supplementation enhanced HG‐induced MFSD2A suppression (mRNA and protein, 1.5 and 5 ng/mL, all *p* < 0.01 vs. HG). PAR2 siRNA transfection abolished CTSS‐mediated MFSD2A suppression (*p* = 0.0177, Figure 7D,E,G). We further excluded the activation effect of endothelial autocrine CTSS on PAR2 (Figure 7H–J). Based on the accumulated evidence, we confirm that CTSS suppresses endothelial MFSD2A expression and regulates vascular permeability through endothelial PAR2 signaling.

**FIGURE 7:**
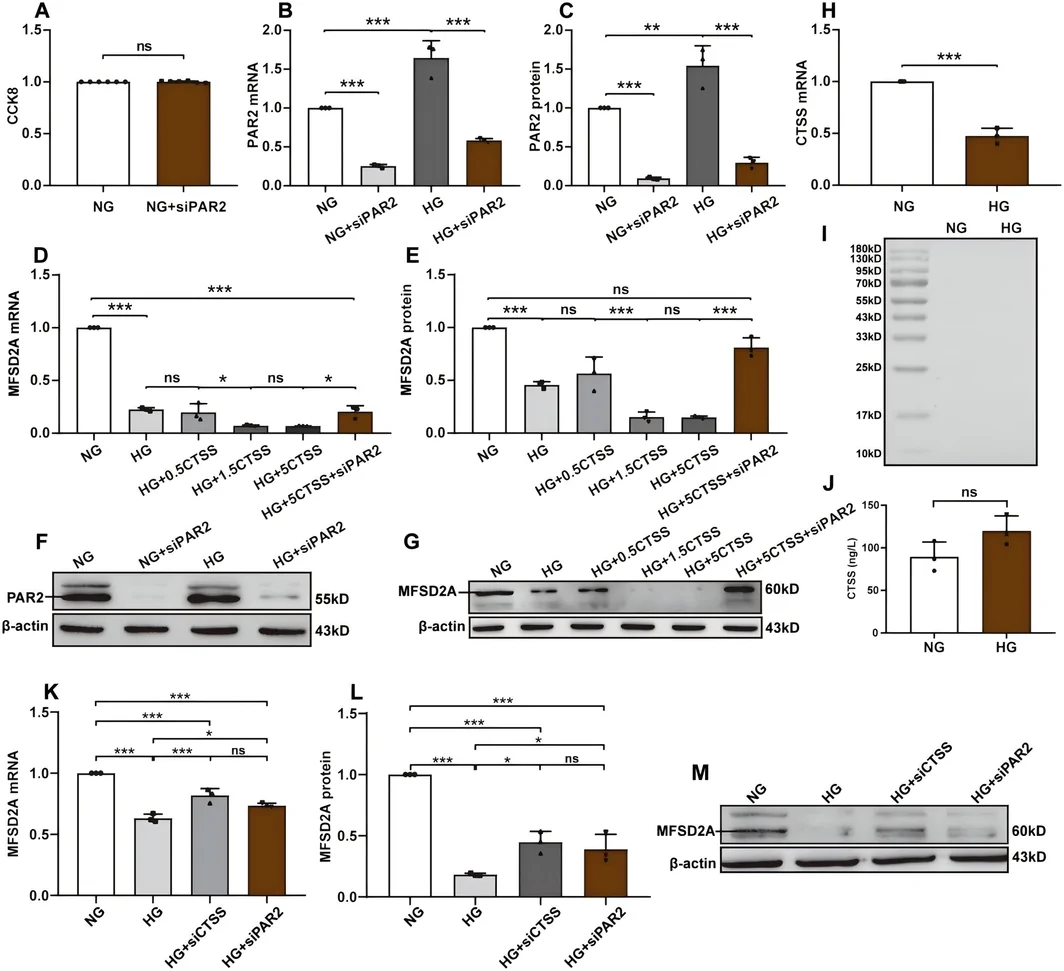
CTSS‐PAR2 mediates microglial–endothelial crosstalk and regulates endothelial MFSD2A expression. (A) CCK8 results of HRMECs (*n* = 6). (B) Relative expression of HRMECs Par2 mRNA (*n* = 3). (C) Relative expression of HRMECs PAR2 protein (*n* = 3). (D) Relative expression of HRMECs Mfsd2a mRNA (*n* = 3). (E) Relative expression of HRMECs MFSD2A protein (*n* = 3). (F) Protein bands of HRMECs PAR2. (G) Protein bands of HRMECs MFSD2A, the concentration gradient of CTSS is 0.5, 1.5, and 5 ng/mL, respectively. (H) Relative expression of HRMECs Ctss mRNA (*n* = 3). (I) Protein bands of HRMECs CTSS. (J) CTSS protein concentration (ng/mL) in HRMECs culture supernatant. (K) Relative expression of HRMECs Mfsd2a mRNA (*n* = 3). (L) Relative expression of HRMECs MFSD2A protein (*n* = 3). (M) Protein bands of HRMECs MFSD2A. Data represents as mean ± SD. ns, no significance. *p < 0.05, **p < 0.01, ***p < 0.001.

To investigate whether microglial‐derived CTSS modulates endothelial MFSD2A via PAR2, we established a microglia‐endothelial transwell co‐culture system under HG condition. HG induced HRMECs MFSD2A suppression (mRNA: *p* < 0.001 vs. NG, protein: *p* < 0.001 vs. NG, Figure 7K–M). Microglial CTSS knockdown (mRNA: *p* = 0.0008 vs. HG, protein: *p* = 0.0118 vs. HG, Figure 7K–M) and endothelial PAR2 knockdown (mRNA: *p* = 0.0282 vs. HG, protein: *p* = 0.0415 vs. HG, Figure 7K–M) both upregulated MFSD2A expression under HG condition. Indicating CTSS‐PAR2 signaling axis regulates endothelial MFSD2A expression during microglia‐endothelial communication. Notably, neither intervention fully restored MFSD2A expression to NG level [HG + siCTSS vs. NG: mRNA (*p* = 0.0009), protein (*p* < 0.001), HG + siPAR2 vs. NG: mRNA (*p* < 0.001), protein (*p* < 0.001)]. Suggesting other MFSD2A regulation exists under diabetic conditions.

## 4 Discussion

DR, a leading microvascular complication of diabetes, remains the primary cause of preventable vision loss and blindness in working‐age populations globally [1]. Epidemiological studies report a global DR prevalence of 34.6% in diabetes [30], underscoring the urgent need to elucidate DR pathogenesis and identify novel therapeutic targets.

### 4.1 Immune Dysregulation in Early DR

Genome‐wide association studies highlight inflammatory pathways as central to DR progression [4]. During preclinical DR, prior to overt vascular abnormalities, immune‐inflammatory imbalance drives retinal structural and functional damage by disrupting barrier integrity and amplifying inflammatory cascades [5]. Microglia, as the retinal intrinsic immune sentinels, regulate immune homeostasis through phagocytic activity and rapid response to extracellular stimuli [31]. Their activation precedes macroglial reactivity, positioning them as early mediators of retinal inflammation [32]. We proved that retinal microglia exhibited morphological activation and redistribution in an early diabetic model, aligning with their proposed role in initial iBRB injury. Similarly, Mills et al. [33] confirmed that diabetic retinas showed elevated fractalkine‐CX3CR1 axis activation, driving microglial–endothelial interactions that induce capillary contraction. The localization of perivascular activated microglia suggests that microglia participate not only in vascular development under physiological conditions, but also that aberrant microglia‐endothelial communication regulates retinal vascular function in pathological contexts [23]. However, the microglial regulation mechanism of retinal immune‐inflammatory homeostasis, along with the molecular pathway mediating aberrant microglia‐endothelial communication in vascular injury, remains to be further elucidated.

### 4.2 CTSS as a Key Inflammatory Mediator

CTSS, a lysosomal cysteine protease upregulated in inflammatory diseases [10], emerged as a critical regulator of microglial polarization. We employed scRNA‐seq to reveal the main function of retinal microglial CTSS and microglia CTSS upregulation in diabetic retina. By establishing microglial CTSS knockdown in diabetic retina, we demonstrated that diabetes drives M1 pro‐inflammatory polarization and increases TNF‐α and IL‐1β expression. These findings align with studies showing microglial M1/M2 dynamics dictate inflammatory resolution or progression in experimental autoimmune uveitis [34]. CTSS further facilitates leukocyte migration via MHC‐II antigen presentation [10, 11, 12]. However, we did not detect obvious infiltration of other peripheral immune cells in retina by using cell‐type annotation of scRNA‐seq and immunohistochemical analysis of various retinal immune markers (CD3, CD20, CD68, CD11b, MPO, Supporting Information 2). Surprisingly, Zhou demonstrated MHC‐II‐dependent Th17 infiltration in uveitis models [35], indicating that DR, as a canonical metabolic disorder disease, the inflammatory cascade triggered by early diabetes may be insufficient to recruit peripheral immune cells for retinal infiltration. Thus, our subsequent research has focused on the retinal resident immune cells, microglia, and their dual role in modulating retinal inflammatory responses and contributing to retinal vascular damage.

### 4.3 PAR2‐MFSD2A Axis in Endothelial Dysfunction

CTSS activates endothelial PAR2 via extracellular cleavage and exacerbates inflammatory injury [29]. Microglia‐endothelial transwell model in our study revealed that microglial CTSS suppressed endothelial MFSD2A through PAR2 signaling. Notably, neither CTSS nor PAR2 knockdown fully restored MFSD2A expression to normal level, implying other regulatory pathways. Kumar corroborated this axis in diabetic nephropathy, where CTSS‐PAR2 inhibition improved vascular outcomes [36]. However, Sayed elucidated high glucose‐induced CTSS upregulation in HUVECs [37]. Our results showed negligible CTSS expression under either normal or high glucose conditions based on HRMECs. This discrepancy likely stems from cell type‐specific responses [22] and methodological differences [38, 39].

### 4.4 MFSD2A: Gatekeeper of Vascular Integrity

BRB breakdown marks DR transition from preclinical to clinical [40]. As the primary cellular constituent of iBRB, endothelial cells directly interface with blood components and serve as frontline responders to systemic metabolic disturbances [22]. Emerging evidence suggested that endothelial barrier permeability is dynamically regulated by transcytosis rather than tight junction integrity in immature retinal vasculature, with CAV‐mediated transcytosis playing a pivotal role [41]. MFSD2A, a microvascular endothelial‐specific suppressor of transcytosis, critically regulates CAV‐dependent transcytosis and endothelial permeability. We identified MFSD2A as an endothelial‐specific transcytosis suppressor and its downregulation in diabetes drove hyperpermeability. scRNA‐seq analyses confirmed MFSD2A dominantly expressed in retinal endothelial cells, MFSD2A overexpression partially restored CAV‐1‐mediated transcytosis without affecting tight junction proteins. Similarly, Wang et al. [8] highlighted the role of MFSD2A in iBRB maintenance through caveolin regulation.

MFSD2A is constitutively expressed in retinal vascular endothelial cells and transports lipids bound to lysophosphatidylcholine into the retina, playing a crucial role in maintaining neural function and vascular permeability [28]. Based on the above function, two drug delivery strategies for the central nervous system are proposed: (1) reversible inhibition of endothelial MFSD2A can enhance the efficiency of transcytosis of macromolecular therapeutics into the retina. (2) Targeted development of small‐molecule drugs conjugated to lysophosphatidylcholine may increase their affinity for endothelial MFSD2A, thereby improving drug delivery efficiency across iBRB [42]. Consequently, MFSD2A‐mediated transcytosis represents a promising therapeutic target for retinal drug development and enhancement of drug bioavailability. Providing a theoretical foundation for ameliorating vision impairments caused by vascular leakage and developing retina‐specific drug delivery approaches.

## 5 Conclusion

Hyperglycemia induces retinal microglial activation and CTSS upregulation, disrupting microglia‐endothelial crosstalk via the PAR2 pathway, suppressing endothelial MFSD2A expression, enhancing transcytosis, compromising iBRB integrity, and accelerating DR progression. Therapeutic strategies targeting microglial CTSS or endothelial MFSD2A represent promising avenues for preserving vision in diabetic patients.

## Author Contributions

C.X. and H.K. designed the experiment. C.X. mainly performed the experiment, C.X. analyzed experimental data, and wrote manuscripts. H.L., Q.X., K.Z., Y.C., Y.W., B.K., and W.Z. assisted in completing the experiment. C.X. and H.K. assisted in revising the manuscript.

## Funding

This work was supported by the National Natural Science Foundation of China (82370827).

## Ethics Statement

The animal study was reviewed and approved by First Affiliated Hospital of Harbin Medical University, ethics approval ID (IACUC number): 2023046.

## Conflicts of Interest

The authors declare no conflicts of interest.

## Supporting information


**Supporting Information: 1.** Establishment of DR mouse model.

**Supporting Information: 2.** Retinal immunohistochemical staining of CD3, CD20, CD68, CD11b, MPO.

**Supporting Information: 3.** Microglia participates in the regulation of retinal immunoinflammatory homeostasis.

**Supporting Information: 4.** Differential expression of Ctss gene between DR and Control group.

**Supporting Information: 5.** Establishment and validation of retinal microglial Ctss knockdown model in diabetic mice.

**Supporting Information: 6.** Retinal microglial proinflammatory phenotype increases in early diabetes and accelerates inflammatory microenvironment.

**Supporting Information: 7.** Immunohistochemical staining of retinal inflammatory factors.

**Supporting Information: 8.** scRNA‐seq profiling reveals retinal endothelial function changes in diabetic retinopathy.

**Supporting Information: 9.** Retinal endothelial MFSD2A overexpression partially rescues diabetes‐induced retinal histopathology.

**Supporting Information: 10.** Dysregulated microglia‐endothelial crosstalk and ultrastructural alterations promote vascular injury in hyperglycemia.

**Supporting Information: 11.** Holographic 3D tomography imaging and 2D co‐culture model between microglia and endothelial cells.

## Data Availability

The article provided the data information during the author's experiment. Further inquiries can be directed to the corresponding author.
