# Supplementary material for: Hyperglycemia‐Induced Microglial CTSS Disrupts Blood‐Retinal Barrier Integrity via PAR2‐Dependent Downregulation of Endothelial MFSD2A
Source: CNS Neurosci Ther. 2026 Sep 28;32(10):e71187. doi: 10.1002/cns.71187 (PMC13618738; doi:10.1002/cns.71187)
Supplement: Supplementary file 1 [file CNS-32-e71187-s002.docx]

**Supplementary Material 8: scRNA-seq profiling reveals retinal endothelial function changes in diabetic retinopathy.** (A) Top 20 DEGs in endothelial cells between DR and Control group (heat map). (B) Enrichment analysis of retinal endothelial-specific high-expressed genes. (C. D) GSEA diagram of DEGs in retinal endothelial cells. Data presents as DR vs. Control, NES>0, *P*<0.05, the gene set "apoptosis" and "type 1 diabetes mellitus" are significantly enriched in DR group.
