# Supplementary material for: Hyperglycemia‐Induced Microglial CTSS Disrupts Blood‐Retinal Barrier Integrity via PAR2‐Dependent Downregulation of Endothelial MFSD2A
Source: CNS Neurosci Ther. 2026 Sep 28;32(10):e71187. doi: 10.1002/cns.71187 (PMC13618738; doi:10.1002/cns.71187)
Supplement: Supplementary file 2 [file CNS-32-e71187-s003.docx]

**Supplementary Material 6: Retinal microglial proinflammatory phenotype increases in early diabetes and accelerates inflammatory microenvironment.** (A) Immunofluorescence staining of retinal M1 (IBA-1+CD86+) phenotype microglia. The white arrows indicate the double positive (IBA-1+CD86+) area. (B) Immunofluorescence staining of retinal M2 (IBA-1+CD206+) phenotype microglia. The white arrows indicate the double positive (IBA-1+CD206+) area. (C) Statistical analysis of immunofluorescence staining of retinal M1 phenotype microglia, the data are presented as the number of double positive (IBA-1+CD86+) area. n=3. (D) Statistical analysis of immunofluorescence staining of retinal M2 phenotype microglia, the data are presented as the number of double positive (IBA-1+CD206+) area. n=3. ns, no significance. **, *P*<0.01.
