# Supplementary material for: Hyperglycemia‐Induced Microglial CTSS Disrupts Blood‐Retinal Barrier Integrity via PAR2‐Dependent Downregulation of Endothelial MFSD2A
Source: CNS Neurosci Ther. 2026 Sep 28;32(10):e71187. doi: 10.1002/cns.71187 (PMC13618738; doi:10.1002/cns.71187)
Supplement: Supplementary file 3 [file CNS-32-e71187-s004.docx]

**Supplementary material 3: Microglia participates in the regulation of retinal immunoinflammatory homeostasis.** (A) Top 20 DEGs in retinal microglia between DR and Control group (heat map). (B) Enrichment analysis of genes highly expressed in microglia (bubble plot). (C. D) GSEA plot of DEGs in retinal microglia. Data presents as DR vs. Control, NES>0, *P*<0.05, the gene set "Cytokine-cytokine receptor interaction" and "TNF signaling pathway" are significantly enriched in DR group.
