# Supplementary material for: Hyperglycemia‐Induced Microglial CTSS Disrupts Blood‐Retinal Barrier Integrity via PAR2‐Dependent Downregulation of Endothelial MFSD2A
Source: CNS Neurosci Ther. 2026 Sep 28;32(10):e71187. doi: 10.1002/cns.71187 (PMC13618738; doi:10.1002/cns.71187)
Supplement: Supplementary file 4 [file CNS-32-e71187-s005.docx]

**Supplementary Material 5: Establishment and validation of retinal microglial Ctss knockdown model in daibetic mice.** GSEA analysis confirmed that retinal microglial Ctss is significantly enriched in the positive regulation of inflammatory response (A), endocytic vesicle (B) and positive regulation of response to external stimulus pathway (C). Data presents as DR vs. Control, NES>0, *P*<0.05, the gene sets are significantly enriched in DR group. (D) Retinal HE staining (×400). NFL: Nerve fiber layer. GCL: Ganglion cell layer. IPL: Inner plexiform layer. INL: Inner nuclear layer. OPL: Outer plexiform layer. ONL: Outer nuclear layer. (E) Immunofluorescence staining of retinal microglia (Iba-1+) and CTSS. The white arrows indicate the double positive (IBA-1+CTSS+) area. (F) Statistical analysis based on the average fluorescence intensity, with data presented as average area of IBA-1+CTSS+. n=3. (G) Protein bands of retinal CTSS. (H) Statistical analysis of protein bands in retinal CTSS. n=3. Data represents as mean ± SD. ns, no significance. *, *P*<0.05. **, *P*<0.01.
