# Supplementary material for: Hyperglycemia‐Induced Microglial CTSS Disrupts Blood‐Retinal Barrier Integrity via PAR2‐Dependent Downregulation of Endothelial MFSD2A
Source: CNS Neurosci Ther. 2026 Sep 28;32(10):e71187. doi: 10.1002/cns.71187 (PMC13618738; doi:10.1002/cns.71187)
Supplement: Supplementary file 5 [file CNS-32-e71187-s006.docx]

**Supplementary Material 1:Establishment of DR mouse model.** (A) Body weight. n=12. (B) Blood glucose. n=12. (C) Plasma insulin. n=12. (D) Daily food consumption (g/day). n=12. (E) Daily water consumption (ml/day). n=12. (F) Statistical analysis of vascular basement membrane thickness (BMT). Retinal BMT presents as the average BMT of five points in each retina. n=3. (G) Statistical analysis of proportion of fluorescein leakage area. Select one image from each of the upper, lower, left, and right quadrants of the retina, the data presents as mean of proportion of retinal leakage area of the four quadrants. n=3. (H) Retinal HE staining images (×400). NFL: Nerve fiber layer. GCL: Ganglion cell layer. IPL: Inner plexiform layer. INL: Inner nuclear layer. OPL: Outer plexiform layer. ONL: Outer nuclear layer. (I) Retinal transmission electron microscopy (TEM) images (×5k). Yellow arrows indicate the thin and smooth Control BM. Green arrows indicate thickening and tortuosity of retinal BM in DR. (J) Vascular permeability results detected by FITC-dextran (×200). Red arrows indicate fluorescence leakage. Data represents as mean ± SD. *, *P*<0.05, ***, *P*<0.001.
