# Supplementary material for: Hyperglycemia‐Induced Microglial CTSS Disrupts Blood‐Retinal Barrier Integrity via PAR2‐Dependent Downregulation of Endothelial MFSD2A
Source: CNS Neurosci Ther. 2026 Sep 28;32(10):e71187. doi: 10.1002/cns.71187 (PMC13618738; doi:10.1002/cns.71187)
Supplement: Supplementary file 6 [file CNS-32-e71187-s008.docx]

**Supplementary Material 9: Retinal endothelial MFSD2A overexpression partially rescues diabetes-induced retinal histopathology.** (A) Retinal HE staining (×400). NFL: Nerve fiber layer. GCL: Ganglion cell layer. IPL: Inner plexiform layer. INL: Inner nuclear layer. OPL: Outer plexiform layer. ONL: Outer nuclear layer. (B) Immunofluorescence staining of retinal endothelial cells (CD31+) and MFSD2A. The white arrows represent the CD31+MFSD2A double positive area. (C) Protein bands of HRMEC MFSD2A. (D) Statistical analysis based on retinal immunofluorescence staining, the data is represented as the total area of CD31+MFSD2A+ double positive area. n=3. (E) Statistical analysis of retinal MFSD2A western blot protein bands. n=3. Data represents as mean ± SD. ns, no significance. *, *P*<0.05. **, *P*<0.01.
