# Supplementary material for: Hyperglycemia‐Induced Microglial CTSS Disrupts Blood‐Retinal Barrier Integrity via PAR2‐Dependent Downregulation of Endothelial MFSD2A
Source: CNS Neurosci Ther. 2026 Sep 28;32(10):e71187. doi: 10.1002/cns.71187 (PMC13618738; doi:10.1002/cns.71187)
Supplement: Supplementary file 7 [file CNS-32-e71187-s009.docx]

**Supplementary Material 7: Immunohistochemical staining of retinal inflammatory factors.** (A) Immunohistochemical staining of retinal IL-1β, TNF-α, IL-10 and TGF-β. (B) Statistical analysis of immunohistochemical staining of retinal IL-1β, TNF-α, IL-10 and TGF-β. n=3. ns, no significance. *, *P*<0.05. ***, *P*<0.001.
