# Supplementary material for: Hyperglycemia‐Induced Microglial CTSS Disrupts Blood‐Retinal Barrier Integrity via PAR2‐Dependent Downregulation of Endothelial MFSD2A
Source: CNS Neurosci Ther. 2026 Sep 28;32(10):e71187. doi: 10.1002/cns.71187 (PMC13618738; doi:10.1002/cns.71187)
Supplement: Supplementary file 8 [file CNS-32-e71187-s010.docx]

**Supplementary Material 2: Retinal immunohistochemical staining of CD3, CD20, CD68, CD11b, MPO.** There was no significant expression difference between DR and Control group.
