# Supplementary material for: Hyperglycemia‐Induced Microglial CTSS Disrupts Blood‐Retinal Barrier Integrity via PAR2‐Dependent Downregulation of Endothelial MFSD2A
Source: CNS Neurosci Ther. 2026 Sep 28;32(10):e71187. doi: 10.1002/cns.71187 (PMC13618738; doi:10.1002/cns.71187)
Supplement: Supplementary file 9 [file CNS-32-e71187-s011.docx]

**Supplementary Material 10: Dysregulated microglia-endothelial crosstalk and ultrastructural alterations promote vascular injury in hyperglycemia.** Heat map of interactions between retinal cell types in Control (A) and DR group (D). Shell diagram of interactions between microglia and other cell types, endothelial cells and other cell types in Control (B.C) and DR group (E.F). The number in shell diagram indicates number of ligand receptor pairs detected between the two indicated cell types. The beginning of the arrow represents the ligand, and the end represents the receptor. (G) Holographic 3D tomography images of microglia-endothelial co-culture. The yellow arrows represent process morphology of microglia under NG conditions, the green arrows represent process morphology of microglia under HG conditions. The red arrows indicate process extended by microglia to establish communication with endothelial cells. The white arrows indicate endothelial cells.
